# *Mycobacterium abscessus* Meningitis Associated with Stem Cell Treatment During Medical Tourism

**DOI:** 10.3201/eid2908.230317

**Published:** 2023-08

**Authors:** Andrew B. Wolf, Kelli M. Money, Arun Chandnani, Charles L. Daley, David E. Griffith, Lakshmi Chauhan, Nathan Coffman, Amanda L. Piquet, Kenneth L. Tyler, Shanta M. Zimmer, Brian T. Montague, Sarah Mann, Daniel M. Pastula

**Affiliations:** University of Colorado School of Medicine, Aurora, Colorado, USA (A.B. Wolf, K.M. Money, A. Chandani, L. Chauhan, N. Coffman, A.L. Piquet, K.L. Tyler, S.M. Zimmer, B.T. Montague, S. Mann, D.M. Pastula);; National Jewish Health, Denver, Colorado, USA (C.L. Daley, D.E. Griffith);; Colorado School of Public Health, Aurora (D.M. Pastula)

**Keywords:** *Mycobacterium abscessus*, meningitis/encephalitis, medical tourism, stem cells, nontuberculous mycobacterium infections, multiple sclerosis, Mexico, bacteria

## Abstract

*Mycobacterium abscessus* infections have been reported as adverse events related to medical tourism. We report *M. abscessus* meningitis in a patient who traveled from Colorado, USA, to Mexico to receive intrathecal stem cell injections as treatment for multiple sclerosis. We also review the management of this challenging central nervous system infection.

*Mycobacterium abscessus* is a rapidly growing nontuberculous mycobacterium (NTM) commonly found in soil and water (*1*). Pulmonary, skin, and soft tissue infections are common nosocomial infections that are often associated with inadequate sterilization of water and reagents (*2*). Rarely, *M. abscessus* can infect the central nervous system (CNS), causing chronic meningitis or abscess, often in the setting of trauma, surgery, or dissemination in immunocompromised hosts (*3*). CNS infections typically manifest as subacute headache, fever, meningismus, or some combination, along with focal neurologic signs if there is an abscess (*3*). *M. abscessus* infection has been reported as a complication of medical tourism (i.e., when patients travel abroad for medical treatment or cosmetic surgeries) (*4*,*5*). *M. abscessus* infections are challenging to treat, requiring prolonged multidrug regimens or surgical intervention (*6*). We present a patient with *M. abscessus* meningitis associated with intrathecal stem cell injections during medical tourism.

## The Case 

This report centers on an immunocompetent woman in her 30s who had been diagnosed with multiple sclerosis (MS) and met 2017 McDonald criteria 3 years before our initial encounter. Her initial MS symptoms were episodic left arm and left leg numbness, and she had multifocal brain, cervical, and thoracic spine demyelinating lesions identified by magnetic resonance imaging. She had never received disease-modifying therapies or other immunosuppressive medications. Results of her baseline neurologic examination was unremarkable.

In October 2022, the patient traveled to a commercial clinic in Baja California, Mexico. During a 4-day visit, she underwent 2 lumbar punctures for intrathecal injection of donor umbilical cord stem cells programmed to treat MS. She pursued treatment at this clinic after reviewing its associated website as part of her research on stem cell treatments for MS.

The day after the second intrathecal injection, she visited an emergency department in the United States for positional headache and received an epidural blood patch for presumed postlumbar puncture cerebrospinal fluid (CSF) leak. She reported nocturnal fevers, but vital signs, neurologic examination, complete blood counts, and computed tomography of the head were unremarkable. She was discharged after her headache improved but subsequently received 2 blood patches in the outpatient setting for recurrent headaches.

Because of persistent fevers, the patient was admitted to an outside hospital 5 days after receiving the third blood patch. At admission, she was febrile (101.3°F), but vital signs and complete blood counts were otherwise within reference ranges and HIV serology results were negative. Neurologic examination remained unremarkable. Sampling of her CSF revealed 74 nucleated cells/μL (76% neutrophils, 20% lymphocytes, 2% monocytes), 64 red blood cells/µL, 84 mg/dL of protein, and 29 mg/dL of glucose (serum glucose 96 mg/dL). Results of herpes simplex virus PCR and enterovirus real-time reverse transcription PCR tests were negative. There was no growth on aerobic or anaerobic bacterial cultures. She received vancomycin and 1 dose of cefepime before changing over to meropenem because rash developed during cefepime infusion. She transitioned to imipenem for a 10-day total course; fever resolved, and headache improved.

Days after completing antibiotics, the patient experienced worsening headache and recurrence of fevers, prompting her admission to our institution. We resampled her CSF and found it contained 104 nucleated cells/μL (50% neutrophils, 42% lymphocytes, 8% monocytes), 3 red blood cells/µL, 47 mg/dL of protein, and 31 mg/dL of glucose (serum glucose 85 mg/dL). We identified 13 CSF-specific oligoclonal bands (reference <2) and noted her IgG index was 1.21 (reference <0.6); those values were consistent with MS but also a potential indicator of CNS infection). We obtained magnetic resonance images of the patient’s brain and cervical, thoracic, and lumbar spine, with and without gadolinium, and found no evidence of active demyelination or infection. We prescribed a course of vancomycin and ceftriaxone as empiric meningitis coverage, and the patient noted improvement of fever and headaches. The patient’s CSF culture became positive for a rapidly growing NTM after 7 days of incubation.

We prescribed the patient a treatment course that included azithromycin (500 mg intravenously [IV] 1×/d), imipenem (500 mg IV 4×/d), and trimethoprim/sulfamethoxazole (5 mg/kg IV 3×/d), tedizolid (200 mg orally 1×/d), and ciprofloxacin (400 mg orally 3×/d), some of which led to intolerable gastrointestinal symptoms. After identifying the NTM as *M. abscessus*, we adjusted the patient’s treatment course on the basis of drug susceptibilities using Clinical and Laboratory Standards Institute cutpoints for resistance (Table 1) (*7*). We stopped trimethoprim/sulfamethoxazole and ciprofloxacin and instituted eravacycline (80 mg IV 1×/d) due to favorable CNS penetration. We initiated ceftaroline (600 mg IV 3×/d) due to synergistic activity with imipenem (*8*). The final treatment regimen (Table 2) brought improvement in headaches and resolution of fevers. In total, it took 8 weeks of evaluations after her initial visit to the outside emergency department to identify and treat *M. abscessus*. Repeat CSF culture after 3 weeks of treatment revealed no growth. The patient has now completed >3 months of treatment without recurrent symptoms.

**Table 1:** *Mycobacterium abscessus* antibiotic susceptibilities for case-patient who underwent intrathecal stem cell injections as treatment for multiple sclerosis at a clinic in Mexico*

Antimicrobial	MIC, μg/mL	Interpretation
Amikacin	64	Resistant
Cefoxitin	32	Intermediate
Ciprofloxacin	≥8	Resistant
Clarithromycin	1	Susceptible
Doxycycline	≥16	Resistant
Imipenem	16	Intermediate
Linezolid	8	Susceptible
Moxifloxacin	≥8	Resistant
Tigecycline	≥8	Resistant
Trimethoprim/sulfamethoxazole	≥8/152	Resistant

*Determined by using Clinical and Laboratory Standards Institute cutpoints for resistance (*7*). There is no recommended cutpoint for tigecycline.

**Table 2:** Final treatment regimen for *Mycobacterium abscessus* meningitis for case patient who underwent intrathecal stem cell injections as treatment for multiple sclerosis at a clinic in Mexico

Drug	Dose
Azithromycin	500 mg IV 1×/d
Ceftaroline	600 mg IV 3×/d
Eravacycline	80 mg IV 1×/d
Imipenem	500 mg IV 4×/d
Tedizolid	200 mg orally 1×/d

*IV, intravenously.

## Conclusions

This patient’s case highlights a serious complication associated with medical tourism. Promising preclinical studies of stem cell treatments has led to stem cell tourism (*9*). A purported ability of stem cell treatments to repair disabling CNS damage has encouraged patients to frequently pursue such treatments, despite the lack of demonstrated efficacy. Without regulatory approval, stem cell treatments are frequently marketed online, with scientific messaging and patient testimonials to project an aura of legitimacy. Such treatments have been linked to serious complications, leading to warnings from the US Centers for Disease Control and Prevention and the US Food and Drug Administration (*10*–*12*).

Peripheral stem cell treatments have been associated with a range of adverse events, including infections and neoplasms (*10*). There is little to ensure the integrity of the manufacturing or administration for such unregulated treatments, and there is no postmarketing surveillance. Inadequate use of sterile technique or use of contaminated water during manufacture or administration of stem cell products may lead to infections. The largest reported incident involved 20 culture-confirmed bacterial infections secondary to donor umbilical cord blood products proposed as treatment for orthopedic conditions (*13*). In addition to infections, neoplastic and inflammatory lesions have been associated with intrathecal stem cell treatments (*9*,*10*).

Diagnosis of *M. abscessus* infection requires isolation of NTM and use of molecular techniques to identify species and subspecies (*1*). *M. abscessus* is difficult to treat becasue of its in vitro antimicrobial resistance and biofilm formation (*1*). The species is generally resistant to typical antituberculous drugs, and those used for prolonged multidrug regimens are often poorly tolerated (*14*,*15*). Drug efficacy is dramatically reduced by the presence in *M. abscessus* of an erythromycin ribosome methylase gene, *erm(41)*, which induces macrolide resistance, or mutational resistance via the 23S ribosomal RNA gene. As such, macrolide companion drugs must be carefully selected (*6*). Regimens to treat CNS *M. abscessus* infection are based on limited evidence because of the rarity of cases and unknown CSF penetrance of many drugs (*3*,*15*). Surgical debridement of abscesses may be necessary.

The literature describes cases of *M. abscessus* infection involving contributory immunosuppression, trauma, or neurosurgery, making our patient’s case rather unique. She has completed >3 months of treatment without recurrent symptoms. In addition to the direct injury from *M. abscessus* meningitis, patients like the one we report face an increased risk of neurologic disability because chronic CNS infection precludes use of immunosuppressive therapies for MS. Therefore, counseling MS patients on the risks of stem cell tourism is fundamental. There is no proven benefit to intrathecal stem cell treatments, and such treatments should be offered only through registered clinical trials. Clinicians should be aware of potential harms of stem cell tourism and report adverse events to public health agencies.
